# Versatile Endogenous Editing of GluRIIA in *Drosophila melanogaster*

**DOI:** 10.3390/cells13040323

**Published:** 2024-02-10

**Authors:** Constantin J. Beckers, Achmed Mrestani, Fabian Komma, Sven Dannhäuser

**Affiliations:** 1 Department of Neurophysiology, Institute of Physiology, University of Würzburg, D-97070 Würzburg, Germany; 2 Department of Neurology, University of Leipzig Medical Center, D-04103 Leipzig, Germany; achmed.mrestani@medizin.uni-leipzig.de; 3 Rudolf Schönheimer Institute of Biochemistry, Division of General Biochemistry, Medical Faculty, Leipzig University, D-04103 Leipzig, Germany

**Keywords:** glutamate receptor, GluRIIA, postsynaptic receptors, postsynaptic receptor field, postsynaptic density, super-resolution imaging, localization microscopy, *d*STORM, synaptic plasticity, homeostasis, homeostatic plasticity, electrophysiology, CRISPR

## Abstract

Glutamate receptors at the postsynaptic side translate neurotransmitter release from presynapses into postsynaptic excitation. They play a role in many forms of synaptic plasticity, e.g., homeostatic scaling of the receptor field, activity-dependent synaptic plasticity and the induction of presynaptic homeostatic potentiation (PHP). The latter process has been extensively studied at *Drosophila melanogaster* neuromuscular junctions (NMJs). The genetic removal of the glutamate receptor subunit IIA (GluRIIA) leads to an induction of PHP at the synapse. So far, mostly imprecise knockouts of the *GluRIIA* gene have been utilized. Furthermore, mutated and tagged versions of GluRIIA have been examined in the past, but most of these constructs were not expressed under endogenous regulatory control or involved the mentioned imprecise *GluRIIA* knockouts. We performed CRISPR/Cas9-assisted gene editing at the endogenous locus of *GluRIIA*. This enabled the investigation of the endogenous expression pattern of GluRIIA using tagged constructs with an EGFP and an ALFA tag for super-resolution immunofluorescence imaging, including structured illumination microscopy (SIM) and *direct* stochastic optical reconstruction microscopy (*d*STORM). All GluRIIA constructs exhibited full functionality and PHP could be induced by philanthotoxin at control levels. By applying hierarchical clustering algorithms to analyze the *d*STORM data, we detected postsynaptic receptor cluster areas of ~0.15 µm^2^. Consequently, our constructs are suitable for ultrastructural analyses of GluRIIA.

## 1. Introduction

Synaptic plasticity is involved in the continuous adaptation of the nervous system to a perpetually changing environment. In the recent decades, synaptic plasticity mechanisms were studied extensively, ranging from the discovery of long-term potentiation in the hippocampus and long-term depression in the cerebellum to homeostatic scaling of synaptic strength and Hebbian plasticity [1,2,3,4]. An important mechanism that stabilizes transmission despite constantly changing environments is presynaptic homeostatic potentiation, which is actively being investigated at glutamatergic NMJs of *Drosophila melanogaster* [5].

The postsynaptic glutamate receptors at *Drosophila* NMJs are obligate heterotetramers composed of the following subunits: GluRIIC, GluRIID and GluRIIE, which are essential components of every glutamate receptor. GluRIIA and GluRIIB functionally compete to be the fourth subunit [6,7]. During PHP, GluRIIA perturbation initially leads to a decrease in the postsynaptic response to the release of a single vesicle [8]. Later, the number of released vesicles homeostatically increases. Therefore, the postsynaptic answer is fully restored [9,10]. Interestingly, the perturbation of GluRIIB does not induce PHP [10]. Apart from the previously described mechanisms of PHP, the abundance of the postsynaptic receptors shows adaptive plasticity too [11,12,13]. How the competition between both receptor types is governed for adaptive modulation remains unclear [7].

In the study of PHP at *Drosophila* NMJs, two *GluRIIA* knockouts have been widely used: the AD9 and the SP16 alleles. Both mutants were generated by p-element-based mutagenesis—a procedure affecting the *GluRIIA* gene itself as well as its environment [8]. In addition to these mutants, GluRIIA constructs were used but were not expressed at the endogenous locus [14,15]. This does not exactly reconstitute the physiological situation because the expression levels of the different glutamate receptor subunits heavily affect the composition of postsynaptic receptor fields [8,10,16,17].

To develop a more beneficial approach for future investigations, we used the clustered regularly interspaced short palindromic repeats (CRISPR)/CRISPR-associated protein 9 (Cas9) system to introduce a genomic editing platform at the endogenous *GluRIIA* locus for φC31-mediated transgenesis [18,19]. Using electrophysiology, we observed that our created *GluRIIA* knockout (*GluRIIA^Δ5555010−5559248^*, subsequently called GluRIIA^KO^) shows the same functional characteristics as the previously used *GluRIIA* knockouts [8]. Furthermore, we demonstrate the versatility of this landing site by endogenously expressing a rescue and two different tagged constructs. This enabled the investigation of the endogenous expression pattern of GluRIIA with different super-resolution imaging modalities. We generated an endogenously expressed GluRIIA fused to the single-domain-antibody tag ALFA [20] (GluRIIA^ALFA^). In the next step, we performed *direct* stochastic optical reconstruction microscopy (*d*STORM) and applied hierarchical clustering algorithms on the generated localization data for an ultrastructural analysis of postsynaptic receptor fields.

## 2. Experimental Procedures

### 2.1. Fly Stocks

Flies were raised on standard cornmeal and molasses medium at 25 °C. *Drosophila melanogaster* male 3rd instar larvae of the following strains were used for experiments:

Wildtype: *w^1118^* (Bloomington Drosophila Stock Center (BDSC), Bloomington, IN, USA).

GluRIIA^KO^ (*TI{attP}GluRIIA^Δ5555010−5559248^*)*: w^1118^; GluRIIA attP DsRed^−^/CyoGFP; +;* (generated in this study) (BDSC#99516).

GluRIIA^rescue^: *w^1118^; GluRIIA attP {Rescue(pCB_02) w^−^}/CyoGFP; +* (generated in this study).

GluRIIA^EGFP^: *w^1118^*; *GluRIIA attP {EGFP(pCB_19) w^−^}/CyoGFP*; *+* (generated in this study) (BDSC#99517).

GluRIIA^ALFA^: *w^1118^*; *GluRIIA attP {ALFA(pCB_20) w^−^}/CyoGFP; +* (generated in this study) (BDSC#99518).

### 2.2. Transgene Construction

All primer sequences are listed in Supplementary Table S1. *Drosophila* embryo injections were conducted by the company Bestgene Inc. (Chino Hills, CA, USA). The plasmids that were used for injection are described in Supplementary Table S2. CRISPR/Cas9-based genome editing was performed as previously described [18]. Plasmids with gRNA for the 5′ CRISPR site (*pCB_3*), 3′ CRISPR site (*pCB_4*) and the associated homology-directed repair (HDR) plasmid (*pCB_2*) were injected into *w^1118^* embryos carrying a germline expressing vas-Cas9 (BDSC#5132) [21]. The original *GluRIIA* locus was replaced with an *attP* landing site and an additional DsRed+ marker for selection. The correct genomic editing was verified by PCR genotyping with primers *cb_43f*/*cb_44r*. Subsequently, the whole edited locus was sequenced via Sanger sequencing. The DsRed+ marker was removed by expressing a germline Cre source. The resulting fly stock TI{attP}GluRIIA^Δ5555010−5559248^ was genotyped with primers *cb_05f*/*cb_57r*, and again followed by Sanger sequencing.

The following plasmids were injected into GluRIIA^KO^ at Bestgene for ΦC31-mediated integration: *pCB_15* (AddgeneID#194756) for GluRIIA^rescue^, *pCB_19* (AddgeneID#194757) for GluRIIA^EGFP^ and *pCB_20* (AddgeneID#194758) for GluRIIA^ALFA^. Integration resulted in a GluRIIA^rescue^, a GluRIIA^EGFP^ and a GluRIIA^ALFA^ stock with additional *w+* selection markers. Integration was verified by PCR genotyping with primers *cb_03f*/*cb_124r* for GluRIIA^rescue^, *cb_137f*/*cb_138r* for GluRIIA^EGFP^ and *cb_139f*/*cb_140r* for GluRIIA^ALFA^. Next, the *white+* transformation marker was again removed by expressing a germline Cre source. The final PCR genotyping was performed using the primer pairs *cb_03f*/*cb_124r* for GluRIIA^rescue^, *cb_137f*/*cb_138r* for GluRIIA^EGFP^ and *cb_139f*/*cb_140r* for GluRIIA^ALFA^. Afterwards, correct editing was verified by sequencing the edited locus.

### 2.3. Fixation, Staining and Immunofluorescence

The procedure for preparation, fixation and staining of the samples was performed as previously reported [22,23,24]. For immunofluorescence imaging of larval NMJs, larvae were dissected in ice-cold hemolymph-like solution (HL-3, [25]), fixed with 4% paraformaldehyde (PFA) in phosphate-buffered saline (PBS) for 10 min and blocked for 30 min with PBT (PBS containing 0.05% Triton X-100, Sigma, St. Louis, MO, USA) including 5% normal goat serum (NGS, Dianova, Hamburg, Germany). Primary antibodies were added and incubated overnight at 4 °C. After two short and three 20 min long washing steps with PBT, the preparations were incubated with secondary antibodies for 3 h at room temperature, followed by two short and three 20 min long washing steps with PBT. The preparations were kept in PBS at 4 °C until imaging. All NMJ data were obtained from abdominal muscles 6/7 in segments A2 and A3.

### 2.4. Structured Illumination Microscopy (SIM)

The preparation, fixation and antibody staining of the samples were performed as described above. Primary antibodies were used at the following concentrations: mouse monoclonal α-GluRIIA, 1:10 (AB_528269, Developmental Studies Hybridoma Bank (DSHB), Iowa City, IA, United States); rabbit-α-GFP, 1:500 (A11122, ThermoFisher, Waltham, MA, USA). Secondary antibodies were used at the following concentrations: goat α-mouse IgGs labeled with Alexa Fluor532, 1:500 (A11002, Thermofisher); goat α-rabbit IgGs labeled with Alexa Fluor488, 1:500 (A-11008, Invitrogen, Waltham, MA, USA). Directly conjugated antibodies were incubated together with secondary antibodies at the following concentrations: goat α-horseradish-peroxidase (α-HRP) IgGs labeled with Alexa Fluor488, 1:500 (123-545-021, Jackson Immuno Research, West Grove, IA, USA); goat α-mouse IgG labeled with Cy5, 1:500 (AB_2338714, Jackson Immuno Research, West Grove, IA, USA); anti-ALFA nanobody labeled with Alexa Fluor647 (FluoTag^®^-X2 anti-ALFA Alexa647, N1502-AF647-L, 15211103, NanoTag Biotechnologies, Göttingen, Germany).

Larval preparations were mounted in Prolong Glass (ThermoFisher) for SIM imaging. Images were acquired at room temperature from NMJs in muscles 6/7 in segments A2 and A3. SIM imaging was performed as previously described [23] using a Zeiss Elyra S.1 structured illumination microscope equipped with an sCMOS camera (pco.edge 5.5 m, Excelitas PCO GmbH, Kelheim, Germany) and an oil-immersion objective (Plan-Apochromat 63x, 1.4 NA, Carl Zeiss, Jena, Germany). Lasers with wavelengths of 488 nm, 531 nm and 641 nm were used. The Z step size was set to 0.1 μm and imaging was performed using five rotations of the grating at five different phase steps. Fourier transformation of the structured illumination images was performed using ZEN 3.4 vsoftware (Carl Zeiss, Jena, Germany) and the subsequent analysis was performed using ImageJ 2.14.0.

### 2.5. Direct Stochastic Optical Reconstruction Microscopy (dSTORM)

Preparations were incubated with primary antibody overnight at 4 °C at the following concentration: mouse monoclonal α-Bruchpilot (Brp) (Brp^Nc82^), 1:100 (AB_2314866, Developmental Studies Hybridoma Bank (DSHB), Iowa City, IA, USA). Goat α-mouse IgGs labeled with Alexa Fluor532 (1:500; A11002, Thermofisher) and NbALFA-Al647 (1:500) were used as secondary antibodies and were incubated with the preparations for 3 h at room temperature. After staining, the larval preparations were incubated in 100 mM mercaptoethylamine (MEA, Sigma-Aldrich, St. Louis, MO, USA) in a 0.2 M sodium phosphate buffer, pH~7.9, to allow the reversible switching of single fluorophores during data acquisition [26]. The configuration of the *d*STORM setup was described previously [23,27]. Briefly, images were acquired using an inverted microscope (Olympus IX-71, 60x, NA 1.49, oil immersion, Hamburg, Germany) equipped with a nosepiece stage (IX2-NPS, Olympus). Alexa Fluor647 and Alexa Fluor532 were excited using 647 nm (F-04306-113, MBP Communications Inc., Montreal, QC, CA) and 532 nm (gem 532, Laser Quantum, Stockport, UKa) lasers. After passing through clean-up filters (BrightLine HC 642/10 and Semrock, ZET 532/10, respectively, Rochester, NY, USA), the laser beams were combined using two dichroic mirrors (LaserMUX BS 514-543 and LaserMUX BS 473-491R, 1064R, F38-M03, AHF Analysentechnik, Tübingen, Germany) and directed onto the probe by an excitation dichroic mirror (HC Quadband BS R405/488/532/635, F73-832, AHF Analysentechnik). The emitted fluorescence was filtered with a quadband filter (HC-quadband 446/523/600/677, Semrock, Rochester, NY, USA) and a long-pass (Edge Basic 635, Semrock) or bandpass filter (Brightline HC 582/75, Semrock) for the red and green channels, respectively, and divided onto two cameras (iXon Ultra DU-897-U, Andor, Oxfordshire, UKa) using a dichroic mirror (HC-BS 640 imaging, Semrock). The final pixel size was 127 nm px^−1^ (red channel) and 130 nm px^−1^ (green channel). A total of 15,000 frames with an exposure time of 10 ms were acquired and single fluorophore localizations were determined using the open-source software rapi*d*STORM (2.1.0) [28]. Only fluorescence spots with an A/D count over 12,000 were analyzed.

### 2.6. Analysis of Localization Data

Localization tables from rapi*d*STORM were analyzed based on previously described algorithms [23,24,27] using custom-written Python code (language version 3.10.5) and the Python interface Jupyter [29]. Regions of interest (ROIs) corresponding to the terminal 6 type Ib boutons, according to the super-resolved Bruchpilot signal (not shown), were marked in reconstructed, binned images (pixel size: 10 nm px^−1^) from rapi*d*STORM using FIJI [30]. Clusters of GluRIIA^ALFA^ localizations were extracted using the Python implementation of hierarchical density-based spatial clustering of applications with noise (HDBSCAN, [31]). The influence of several settings of the main free parameters and all combinations thereof (minimum cluster size: 10–100 in increments of 10; minimum samples: 2, 5–25 in increments of 5 and 30–100 in increments of 10) on the number of detected clusters per image was tested. The final analysis parameters (minimum cluster size = 40, minimum samples = 10) were set in the stable range that yielded most plausible clustering results. Cluster areas were determined by computing 2D alpha shapes in Python CGAL (Computational Geometry Algorithms Library, [27]). Expectedly, increasing alpha values yielded increasingly larger cluster areas (tested parameters: alpha = x^2^ nm^2^ with x ranging from 5 to 200 in increments of 5). The final quantification was performed with alpha = 9025 nm^2^, the first parameter that yielded a percentage cluster area increase per step below 5%. Exclusion criteria for the ultimately presented GluRIIA^ALFA^ clusters were area ≤0.03 µm^2^ and ≥0.3 µm^2^, according to a previous quantification of presynaptic active zones [27].

### 2.7. Electrophysiology

TEVC recordings (Axoclamp 2B amplifier, Digidata 1440A; Molecular Devices, San José, CA, USA) were obtained from abdominal muscle 6 in segments A2 and A3 as previously described [22,23,24]. All measurements were obtained at room temperature in HL-3 [25] with the following composition (in mM): NaCl 70, KCl 5, MgCl_2_ 20, NaHCO_3_ 10, trehalose 5, sucrose 115, Hepes 5, and CaCl_2_ 1, pH adjusted to 7.2. Intracellular electrodes had resistances of 10–30 MΩ and were filled with 3 M KCl. For the analysis, only cells with an initial membrane potential of at least −50 mV and a membrane resistance of ≥4 MΩ were included. During recordings, cells were clamped at a holding potential of −80 mV (miniature EPSCs) or −60 mV (evoked EPSCs). To evoke synaptic currents, nerves were stimulated via a suction electrode with pulses with a 300 μs length and typically at 5–12 V (Grass S48 stimulator and isolation unit SIU5; Astro-Med, West Warwick, RI, USA). Signals were low-pass filtered at 10 kHz and analyzed in Clampfit 11.1 (Molecular Devices, San José, CA, USA). Paired-pulse recordings were performed with interstimulus intervals of 30 ms. Between recordings, cells were given a 10 s rest. For the analysis, 5–10 traces per interval were averaged. To assess the basal synaptic transmission, 10 EPSCs evoked at 0.2 Hz were averaged per cell. The quantal content was estimated by dividing the mean evoked excitatory postsynaptic current (eEPSC) amplitude by the mean miniature excitatory postsynaptic current (mEPSC) amplitude measured in the same cell. Therefore, mEPSCs recorded at a −80 mV holding potential were scaled to −60 mV assuming a reversal potential of 0 mV and a linear current voltage relationship [32,33].

### 2.8. Statistics

Statistical analyses were performed with SigmaPlot 13 (v13.0, Systat Software) or GraphPad Prism 9 (San Diego, CA, USA). The D’Agostino–Pearson Test or Shapiro–Wilk test was used to test normality. If the data were normally distributed, they were reported as the mean ± SEM unless indicated otherwise. Data not following a normal distribution were reported as the median with 25th and 75th percentiles. The “n” denotes the sample number. In box plots, horizontal lines represent the median, boxes the quartiles and whiskers the 10th and 90th percentiles. Scatter plots show individual data points unless indicated otherwise. Bin counts in histograms were normalized to the total number of observed events which was set to 1. All plots were generated using SigmaPlot (v13.0) or Prism (10.0.3). Figures were assembled using Adobe Illustrator (27.4.1). Supplementary Tables contain all numerical values not stated in the text and figure legends including *p*-values and samples sizes.

### 2.9. Code and Data Availability

The authors declare that custom-written Python code and all data sets supporting the findings of this work are available from the corresponding authors.

## 3. Results

### 3.1. Creation of a Genomic Editing Platform at the GluRIIA Locus

**Figure 1:**
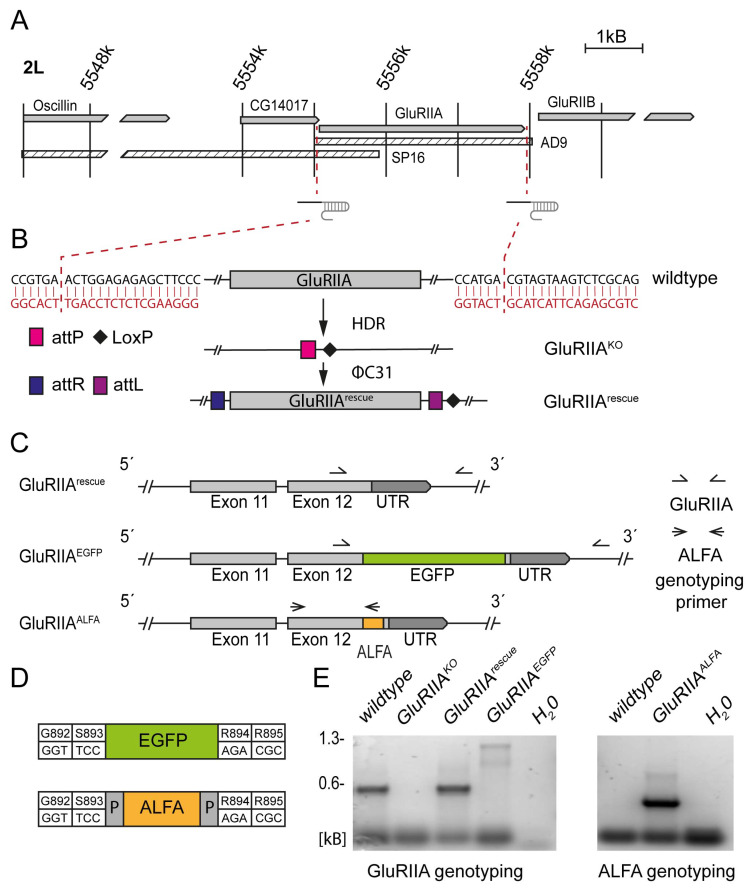
Creation of an endogenous editing platform at the *GluRIIA* locus. (**A**) Map of the *GluRIIA* locus and the adjacent region. Hatched boxes represent two widely used null alleles (SP16 and AD9). Red lines indicate Cas9 cut sites. (**B**) Integration mediated by ΦC31 into *attP* landing site for GluRIIA^rescue^. (**C**) Tags in GluRIIA^EGFP^ and GluRIIA^ALFA^ were integrated into exon 12. Arrows highlight two different primer pairs for PCR genotyping. (**D**) EGFP and ALFA tags were inserted between serine 893 and arginine 894. The ALFA tag is enclosed by a proline on each side. (**E**) Agarose gel electrophoresis of PCR products from genotyping of wildtype, GluRIIA^KO^, GluRIIA^rescue^, GluRIIA^EGFP^ and GluRIIA^ALFA^ with GluRIIA and GluRIIA^ALFA^ genotyping primer pairs to confirm correct editing. H_2_O probe used as control.

The *GluRIIA* gene is located on the second chromosome, directly adjacent to *GluRIIB*. Previous knockout mutants like the AD9 and SP16 alleles were created by p-element-based mutagenesis and affect a region larger than the *GluRIIA* locus [8]. For example, the CG14017 and Oscillin genes are deleted in the SP16 null mutant (Figure 1A). Here, we chose a previously described CRISPR/Cas9-based editing approach [18] to enable the precise manipulation of *GluRIIA*. Thus, a genetic editing platform for φC31-mediated transgenesis was established at the endogenous genetic locus (Figure 1A,B). To demonstrate versatility of this editing platform, different constructs were created: a genomic rescue (GluRIIA^rescue^) and two tagged versions of the GluRIIA subunit—one construct with EGFP (GluRIIA^EGFP^) and one with an ALFA tag (GluRIIA^ALFA^). Both tags’ coding sequences were integrated into the last exon (exon 12) of the *GluRIIA* gene (Figure 1C) between serine 893 and arginine 894 (Figure 1D), corresponding to a previously established location in the C-terminal tail of the receptor subunit that does not interfere with the physiological properties [14]. The ALFA tag has been enclosed by additional prolines on each side to reduce the potential influence of adjacent secondary structures [20]. The correct integration of the constructs was confirmed by PCR genotyping (Figure 1C,E).

### 3.2. Expression of the GluRIIA Subunit at Larval Drosophila NMJs

To assess the expression of the endogenously tagged GluRIIA subunits, we performed structured illumination microscopy (SIM). To identify *Drosophila* larval NMJs, we used an anti-HRP antibody. To test for the presence of GlurIIA itself as well as the presence of its EGFP or ALFA tag, we also added an anti-GluRIIA and anti-GFP or anti-ALFA antibody, respectively.

**Figure 2:**
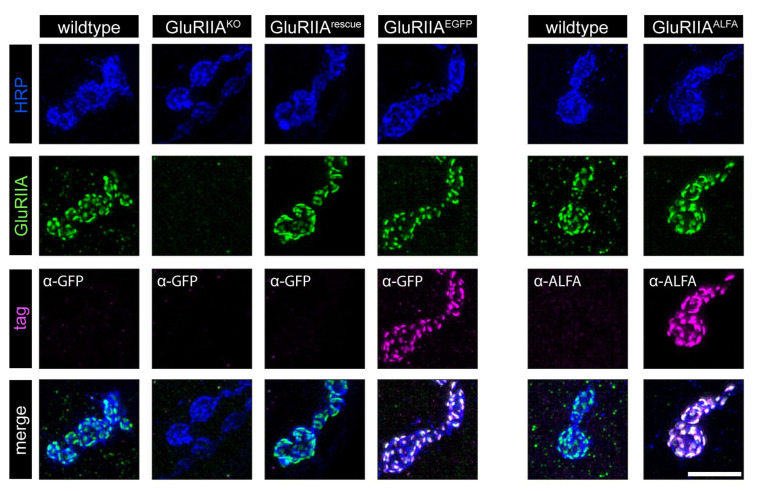
Structured illumination microscopy of *Drosophila* larval NMJs in wildtype and GluRIIA mutants. Immunostaining of NMJs at muscles 6/7 in wildtype and GluRIIA mutant (GluRIIA^KO^, GluRIIA^rescue^, GluRIIA^EGFP^ and GluRIIA^ALFA^) *Drosophila melanogaster* male 3rd instar larvae. Staining was performed with antibodies against HRP, the GluRIIA subunit (anti-GluRIIA) and the inserted tag (anti-GFP or anti-ALFA). Scale bar: 5 μm.

Anti-GluRIIA staining could be detected in wildtype, GluRIIA^rescue^, GluRIIA^EGFP^ and GluRIIA^ALFA^ NMJs, whereas no signal could be observed in our newly created GluRIIA^KO^ (Figure 2). After demonstrating the presence or absence of the GluRIIA subunit, we tested the tags in GluRIIA^EGFP^ and GluRIIA^ALFA^. Both tags exhibited a strong expression at the NMJ (Figure 2), enabling further investigation at the nanoscale level.

### 3.3. Induction of Presynaptic Homeostasis at GluRIIA Mutant NMJs and Normal Function in Endogenously Tagged Constructs

**Figure 3:**
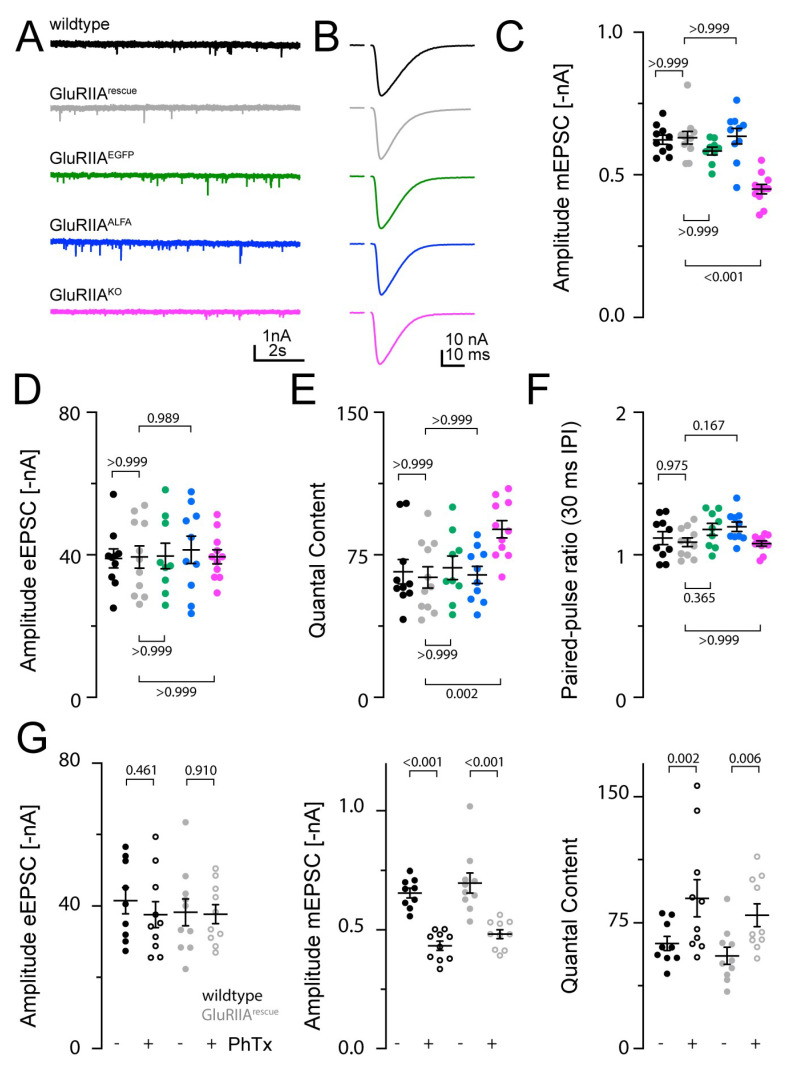
Electrophysiological characterization of the newly generated GluRIIA mutants. (**A**) Representative traces of miniature excitatory postsynaptic currents (mEPSCs) recorded in 1 mM Ca^2+^ at wildtype (black), GluRIIA^rescue^ (gray), GluRIIA^EGFP^ (green), GluRIIA^ALFA^ (blue) and GluRIIA^KO^ (magenta) NMJs. (**B**) Representative traces of evoked EPSCs (eEPSCs) recorded in 1 mM extracellular Ca^2+^ at NMJs for the same four genotypes. (**C**) mEPSC amplitude (mean ± SEM) of each genotype. Scatter plots show individual data points; individual p-values are indicated. (**D**) Evoked excitatory postsynaptic current (eEPSC) amplitude, quantal content (**E**) and paired-pulse ratios with 30 ms interstimulus interval (**F**) for the four tested genotypes. (**G**) eEPSC amplitude (left), mEPSC amplitude (middle) and quantal content (right) in wildtype (black) and GluRIIA^rescue^ (gray) animals treated with PhTx in DMSO (+, open circles) or DMSO alone (−, filled circles). GluRIIA^rescue^ larvae still exhibited presynaptic homeostatic potentiation in response to PhTx stimulation.

To investigate the functionality of our receptor constructs, we applied the two-electrode voltage clamp (TEVC) technique to muscle 6 at segments A2/A3 of 3rd instar larvae (Figure 3). We started with recording spontaneous postsynaptic currents, which correspond to the postsynaptic response to a single presynaptic vesicle (Figure 3A). The mEPSCs in GluRIIA^KO^ showed a significant reduction in amplitude (more than 25% compared to wildtype; numerical data are presented in the supplementary material). The rescue construct and both tagged constructs rescued this phenotype and show comparable mEPSC sizes to wildtype (Figure 3C). In the next step, we measured eEPSCs (Figure 3B). No significant differences concerning the eEPSC amplitude could be observed compared to wildtype (Figure 3D). Consequently, the calculated quantal content of GluRIIA^rescue^, GluRIIA^EGFP^ and GluRIIA^ALFA^ was comparable to that of wildtype. However, the quantal content was increased in GluRIIA^KO^ animals (Figure 3E). This might translate into an increase in vesicle release that was previously observed for chronic PHP [33]. Next, we analyzed the short-term synaptic plasticity and found unchanged paired pulse ratios (30 ms interpulse interval (IPI)) across all constructs (Figure 3F).

Having confirmed the induction of chronic PHP in GluRIIA^KO^, we next turned to acute PHP. Therefore, we used the polyamine philanthotoxin 433 (PhTx) to block postsynaptic GluRIIA (Figure 3G). This pharmacological perturbation of GluRIIA led to a significant decrease in mEPSC size in wildtype as well as GluRIIA^rescue^. As expected, the quantal content after PhTx treatment was significantly increased in wildtype and GluRIIA^rescue^. Thus, wildtype and GluRIIA^rescue^ demonstrated comparable acute PHP reactions.

### 3.4. Determining the Size of Postsynaptic Receptor Fields Using Single-Molecule Localization Microscopy

**Figure 4:**
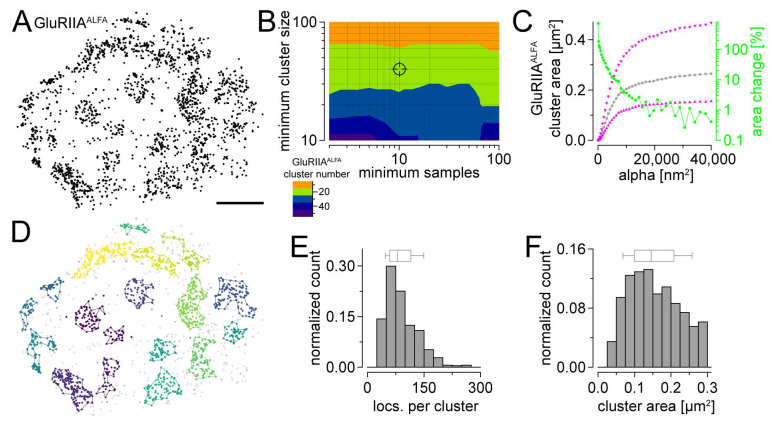
ALFA tagging permits quantification of GluRIIA receptor fields using single-molecule localization microscopy. (**A**) Scatter plot of GluRIIA^ALFA^ localizations (black) at a representative type Ib bouton of abdominal muscles 6/7 of a male 3rd instar *Drosophila* larva. The localizations were obtained by single-channel *direct* stochastic optical reconstruction microscopy (*d*STORM) using Alexa Fluor647-conjugated single-domain FluoTag^®^-X2 anti-ALFA antibody. (**B**) Contour plot displaying the median number of GluRIIA^ALFA^ clusters per image (n = 24 from 10 animals) depending on hierarchical density-based spatial clustering (HDBSCAN) parameters “minimum samples” (x axis) and “minimum cluster size” (y axis). Crosshair indicates the parameter combination (minimum cluster size = 40, minimum samples = 10) which was chosen for further analyses in (**C**,**D**). (**C**) Line and scatter plots of median GluRIIA^ALFA^ cluster area (gray, n = 924 GluRIIA^ALFA^ clusters from 24 NMJs and 10 animals) as well as 25th and 75th percentiles (up- and downward magenta triangles, respectively) plotted against the alpha values which were used for the determination of alpha shape areas. The green line and scatter plots indicate the percent increases of cluster areas with increasing alpha. This relative increase dropped below 5% at an alpha value of 9025 nm^2^ (used for further analyses in (**D**–**F**)). (**D**) Localizations from (**A**) after HDBSCAN-based cluster extraction with different colors for different clusters. Unclustered localizations are shown as gray dots. Colored lines display alpha shapes used for area quantification. (**E**) Number of localizations per GluRIIA^ALFA^ cluster shown as histogram and box plot, where box indicates the median and 25th and 75th percentiles and whiskers the 10th and 90th percentiles (n = 625 GluRIIA^ALFA^ clusters from 24 NMJs and 10 animals). (**F**) GluRIIA^ALFA^ cluster area shown as histogram and box plot. Scale bar in (**A**): 1 µm.

Finally, we tested the applicability of the GluRIIA^ALFA^ line for single-molecule localization microscopy (SMLM). We used a commercially available, directly Alexa Fluor647-coupled single-domain antibody directed against the epitope tag and performed *direct* stochastic optical reconstruction microscopy (*d*STORM) (Figure 4A). A previously described hierarchical density-based spatial clustering of applications with noise (HDBSCAN)-based analysis [23,24,27] was applied to extract and quantify the GluRIIA^ALFA^ clusters. After determining the optimal parameters for cluster detection (Figure 4B) and area quantification using alpha shapes (Figure 4C,D), we analyzed the distribution of the number of localizations and area per GluRIIA^ALFA^ cluster (Figure 4E,F). The extracted clusters contained ~80 localizations (Figure 4E). Strikingly, we obtained a median area of ~0.15 µm^2^ for postsynaptic GluRIIA fields (Figure 4F) which is ~25% larger than previously described measures of presynaptic active zones (~0.11–0.12 µm^2^, [27,34]). In summary, we demonstrated the first application of the ALFA tag combined with single-domain antibodies for SMLM in *Drosophila*, revealing a further experimental approach provided by our newly generated editing platform.

## 4. Discussion

Ionotropic glutamate receptors are a major player in synaptic transmission and synaptic plasticity [35]. Defects in glutamatergic transmission are involved in several human ailments like Alzheimer’s disease and glaucoma [36,37]. *Drosophila* NMJs have been utilized for decades to conduct research on the role of glutamate receptors in synaptic transmission and in diverse forms of synaptic plasticity [5,38]. *Drosophila* and larval NMJs as a model for glutamatergic transmission exhibits several advantages. A vast number of methods for genetic manipulation are available. Combined with the short generation time, this enables the fast expression of transgenic constructs [39]. Furthermore, the NMJs of *Drosophila* are accessible to several electrophysiological methods, ranging from patch clamp analysis to TEVC [14,32,40]. This accessibility enabled the study of several aspects of glutamate receptor physiology in the past like activation, reactivation and desensitization kinetics [41,42]. In recent years, several studies showed the suitability of *Drosophila* NMJs for super-resolution imaging like *d*STORM, which enables studies of the ultrastructure of a synapse and its changes during plasticity [27]. To facilitate future research on GluRIIA physiology and to circumvent the limitations of past approaches, we created a genetic editing platform at the *GluRIIA* locus. Utilizing a CRISPR/Cas9-based approach, an *attP* site at the endogenous locus of *GluRIIA* was established [18] that provides a precisely defined knockout of this subunit. Other CRIPSR/Cas9-based knockouts of *GluRIIA* were described recently [43]. In contrast to this approach, our method enables the versatile expression of diverse genetic constructs under the endogenous promoter via ΦC31-mediated integration.

We utilized the newly generated editing platform and created a rescue construct of the GluRIIA subunit as well as two tagged versions. In addition to an EGFP tag, an ALFA tag for camelid single-domain antibodies was introduced [20]. This approach reduces the linkage error which is caused by the combination of primary and secondary antibodies and therefore improves the spatial resolution [44,45,46,47]. Our tagged versions of the GluRIIA subunit do not differ in their electrophysiological properties from the wildtype subunit and their strong expression enables super-resolution imaging.

The expression level of GluRIIA and its regulation have a direct influence on the postsynaptic receptor field and mEPSC size [10,16]. However, previously used tagged GluRIIA mutants were not expressed endogenously [14,15,48]; thus, our GluRIIA constructs represent a more suitable situation.

Mechanistical differences between chronic and acute forms of PHP have been increasingly discovered [49,50,51]. For the induction of chronic homeostasis, the physical loss of the C-terminal tail of GluRIIA plays a fundamental role [52]. However, the precise mechanism of acute PHP induction remains elusive. The application of PhTx [53] to wildtype and our rescue construct revealed the possibility of inducing a comparable acute PHP reaction for both genotypes. In addition to their role in presynaptic plasticity, the postsynaptic receptor field itself shows mechanisms of adaptive plasticity [13]. To conduct further research on the receptor field’s ultrastructure and its changes during plasticity, super-resolution immunofluorescence imaging techniques are a promising tool. To illustrate the suitability of our receptor constructs, we utilized our ALFA-tagged GluRIIA subunit for *d*STORM imaging and calculated the size of the postsynaptic receptor clusters—a parameter which influences synaptic transmission efficiency [54]. Interestingly, the GluRIIA cluster sizes appeared to be ~25% larger compared to presynaptic Brp-positive active zones [27]. This size difference between the postsynaptic GluRIIA clusters compared to the presynaptic clusters could contribute to the efficient glutamate capture at the postsynapse. Furthermore, our calculated cluster size is larger than recently published values [55]. This is probably due to fundamentally different imaging techniques and the data analysis.

## 5. Conclusions

Here, we established a versatile genetic editing platform at the *GluRIIA* locus, a gene that is implicated in different forms of synaptic plasticity at *Drosophila* NMJs, facilitating the expression of a construct of interest under endogenous regulatory control. We created a precise knockout of *GluRIIA* as well as a corresponding rescue and two tagged versions—one with an EGFP tag and one with an ALFA tag. The rescue construct and both tags do not disturb the receptor’s function and the ALFA tag is suitable for super-resolution imaging. In future studies, the newly generated transgenesis platform will be useful in investigating different modalities of receptor function, e.g., by targeting a calcium or a glutamate sensor to the postsynaptic density or by performing targeted mutagenesis of GluRIIA.

We provide our newly generated *Drosophila* strains and the needed reagents to the scientific community to conduct further research elucidating the function of GluRIIA in pre- and postsynaptic forms of synaptic plasticity.

## Data Availability

The raw data supporting the conclusions of this article will be made available by the authors on request.

## Supplementary Materials

The following supporting information can be downloaded at https://www.mdpi.com/article/10.3390/cells13040323/s1, Table S1: Primer sequences; Table S2: Plasmids generated and used in this study; Table S3: Electrophysiological analysis of spontaneous and evoked synaptic transmission; Table S4: Statistical comparison of spontaneous and evoked synaptic transmission; Table S5: Electrophysiological analysis of acute presynaptic homeostasis; Table S6: Statistical comparison of acute presynaptic homeostasis in wildtype and GluRIIA^rescue^ animals; Table S7: *d*STORM analysis of GluRIIA^ALFA^.
