# Supplementary material for: Versatile Endogenous Editing of GluRIIA in Drosophila melanogaster
Source: Cells. 2024 Feb 10;13(4):323. doi: 10.3390/cells13040323 (PMC10887371; doi:10.3390/cells13040323)
Supplement: Supplementary file 1 [file cells-13-00323-s001.zip › cells-2850372-supplementary.pdf]

SUPPLEMENTARY MATERIAL

**Primer ID**	**5'-3' sequence**
cb_03f	GCGCATTTGGGTATATTGAATG
cb_04r	GCCAGAAGAACCAGGAACTGTAG
cb_05f	CCGAAGAGCATCCGTTGTGC
cb_06r	CTATAATCCCCTCCAGAAACCC
cb_07f	CGTTCACCTGCGAATTCGCGAGATGTAGTCATTGTAATTG
cb_08r	AACGCACCTGCATTCCTACTCACGGCGGAGAGTCCGAGT
cb_09f	GCTGCCTAGGCGTAGTAAGTCTCGCAGAGA
cb_10r	ATGCCCTAGGAGATGTCCGAGAAGGCGGCG
cb_11f	AGTCGAATTCACTGGAGAGAGCTTCCCATC
cb_12r	GACTGGTACCTCATGGAGAACAGCAGCCAT
cb_13f	CTTCGGGAAGCTCTCTCCAGTTCA
cb_14r	AAACTGAACTGGAGAGAGCTTCCC
cb_15f	CTTCGCTGCGAGACTTACTACGTCA
cb_16r	AAACTGACGTAGTAAGTCTCGCAGC
cb_43f	GAGTTCTCTCAGTTGGGGGCGT
cb_44r	ATGATCTAGAGTCGCGGCCCC
cb_45f	TCATGAAACTGGCAGCCAGGGA
cb_46r	GGGCAGCTGCACGGGCTTCTT
cb_47f	AAGGGCGAGATCCACAAGGCC
cb_48r	GCCAGAAATCGGCCAGATCCG
cb_49f	GTAGCCCAGCTGGACGATGGTC
cb_50r	GCGAAGCACAAACCCTTGACGG
cb_51f	CCAAGGGCTTCAATATCTCCGGC
cb_52r	GCCCCCAACTGAGAGAACTCAAAG
cb_53f	CGCAGAGATCGCTCGTGGCG
cb_54r	TCATCCCCTCGTTCGAATTCCTGC
cb_57r	CGAAGTTATCTACGCCCCCAAC
cb_62r	ACGGGAACTCCTCTCGTAGGC
cb_66f	CGCCTGCAGGATCTGATGAA
cb_67r	CTGAGACCTAGGCAGAATGTCG
cb_68f	TGGTTGCCACCTAATCCCGCC

cb_69r	TGCAGACACGAGCATTAGCTGG
cb_90f	ACTATACAAAAGACGCAGCTCCAAGGAAAAG
cb_91r	TCCTATGAGCGGAACCGGATGATCGCCTG
cb_92f	ATCCGGTTCCGCTCATAGGAGTAAAGGAG
cb_93r	AGCTGCGTCTTTTGTATAGTTCATCCATGC
cb_102f	GTGGGCTCCATCATGCAACAGGG
cb_104r	TCTGCGTACGATAACTTCGTATAGCATACAT
cb_106r	TCACAGGTGACATTGTGGTTCTTCCACCAC
cb_107f	AACCACAATGTCACCTGTGACTCCTACC
cb_108r	ACTCTCTAATGAAATGCCAAAGTAGAAGAC
cb_109f	TTGGCATTTCATTAGAGAGTCGCATTGG
cb_115r	TCATCCGCATAGTCTGCCAAA
cb_118f	CGGAATATTGGACGATTGGC
cb_124r	ACTAGTGCTCTTCTATAACTTCG
cb_137f	ACCTGTGACTCCTACCACGA
cb_138r	TCACCTTCACCCTCTCCACT
cb_139f	CCGGACCAACCTCAGCGTGT
cb_140r	CGGCGCAGCTCCTCCTCCA
cb_145f	CTCATCTTTGCCCTGAAGTTTTG
cb_146r	CATTAGCTGGAAATGGACCAG
cb_147f	TCACTGGAGAGAGCTTCCCA
cb_148r	GCGTCGACCACTTTCAATCG
cb_151f	CAACGTTTTGCCCTCGAAGG
cb_152r	GCAACATGGTTTGGTTCCCC

Supplementary Table 1. Primer sequences. All primers which have been used in this study are listed.

Plasmid ID	Description
pCB_02	GluRIIA-HDR: A PCR-fragment for the 5´ homology arm was created with primer pair *cb_07f*/*cb_08r*. This fragment was digested with *Aar*I and ligated into an *Aar*I-fragment of *pHD-DsRed-attP* (*pTL620*), thereby creating *pCB_01*. For the 3´ homology arm, a PCR-fragment was created with primer pair *cb_09f*/*cb_10r*. This fragment was digested with *Avr*II and then ligated into an *Avr*II-fragment of *pCB_01*, thereby creating the final plasmid *pCB_02*.
pCB_03	pU6-gRNA(5'): gRNA-target-site for the 5´ cleavage site was identified by utilizing the CRISPR Optimal Target Finder (Gratz et al., 2014). Cas9 cleavage site was verified by sequencing the PCR product *cb_05f*/*cb_06r*. Specific sequences for the 5` gRNA (GGGAAGCTCTCTCCAGTTCACGG) were ordered as 5´-phosphorylated oligonucleotides (*cb_13f*/*cb_14r*). These were annealed and then ligated into the *Bbs*I sites of the *pU6-BbsI-chiRNA* vector, thereby creating *pCB_03*.
pCB_04	pU6-gRNA(3'): gRNA-target-site for the 3´ cleavage site was identified by utilizing the CRISPR Optimal Target Finder. Cas9 cleavage site was verified by sequencing a PCR product (*cb_03f*/*cb_04r*). Specific sequences for the 3` gRNA(CTGCGAGACTTACTACGTCATGG) were ordered as 5´-phosphorylated oligonucleotides (*cb_15f*/*cb_16r*). These were annealed and then ligated into the *Bbs*I sites of the *pU6-BbsI-chiRNA* vector, thereby creating *pCB_04*.
pCB_15	attB-GluRIIA-Rescue: A 4,3 kb fragment was generated from *w1118* genomic DNA with primer pair *cb_11f*/*cb_12r*. In the following, digestion with *EcoR*I/*Kpn*I and ligation into a *EcoR*I/*Kpn*I fragment of *pGE-attB-GMR*, thereby creating the rescue plasmid *pCB_15*. This plasmid is available at addgene (catalog # 194756).
pCB_19	attB-GluRIIA-EGFP: A 0,7 kb PCR product was generated from an EGFP-containing plasmid (*pAM_46*) with primer pair *cb_92f*/*cb_93r*. Two fragments adjacent to the final genomic location of the EGFP tag were created with primer pair *cb_102f*/*cb_91r* (5´) and *cb_90f*/*cb_104r* (3`). All three fragments were combined to one

	product by utilizing the NEB HIFI-DNA-Assembly kit and then multiplicated via PCR with primer pair *cb_102f*/*cb_104r*. The resulting PCR product was digested with *Avr*II/*BsiW*I and ligated into a *Avr*II/*BsiW*I fragment of *pCB_15*. This resulted in the creation of the GluRIIA-EGFP plasmid *pCB_19* (available at addgene, catalog # 194757).
pCB_20	attB-GluRIIA-ALFA: A PCR product containing the sequence for the ALFA tag was generated from an ordered plasmid (*pCB_18*) using primer pair *cb_107f*/*cb_108r*. Two fragments adjacent to the final genomic location of the ALFA tag were created with primer pair *cb_102f*/*cb_106r* (5´) and *cb_109f*/*cb_104r* (3`). All three products were combined to one product by utilizing the NEB HIFI-DNA-Assembly kit and then multiplicated via PCR with primer pair *cb_102f*/*cb_104r*. The resulting PCR product was digested with *Avr*II/*BsiW*I and ligated into an *Avr*II/*BsiW*I fragment of *pCB_15*. This resulted in the creation of the GluRIIA-ALFA plasmid *pCB_20* (available at addgene, catalog # 194758).

Supplementary Table 2. Plasmids generated and used in this study. All plasmids, created and used in this study are listed and described.

parameter	wildtype	GluRIIA^{rescue}	GluRIIA^{EGFP}	GluRIIA^{ALFA}	GluRIIA^{KO}
**mEPSC amplitude [-nA]**	0.623 ± 0.016 n = 10 NMJs, 8 animals	0.631 ± 0.022 n = 11 NMJs, 7 animals	0.584 ± 0.014 n = 9 NMJs, 7 animals	0.636 ± 0.027 n = 10 NMJs, 6 animals	0.450 ± 0.017 n = 11 NMJs, 8 animals
**eEPSC amplitude [-nA]**	39.00 ± 2.66 n = 10 NMJs, 8 animals	39.40 ± 3.12 n = 11 NMJs, 7 animals	39.70 ±3.58 n = 9 NMJs, 7 animals	41.42 ± 3.82 n = 10 NMJs, 6 animals	39.47 ± 1.96 n = 11 NMJs, 8 animals
**PPR (30 ms IPI)**	1.117 ± 0.045 n = 10 NMJs, 8 animals	1.089 ± 0.030 n = 11 NMJs, 7 animals	1.179 ± 0.041 n = 9 NMJs, 7 animals	1.198 ± 0.033 n = 10 NMJs, 6 animals	1.080 ± 0.018 n = 11 NMJs, 8 animals
**quantal content**	66.19 ± 6.40 n = 10 NMJs, 8 animals	63.20 ± 5.56 n = 11 NMJs, 7 animals	68.22 ± 6.16 n = 9 NMJs, 7 animals	64.46 ± 4.48 n = 10 NMJs, 6 animals	88.43 ± 4.55 n = 10 NMJs, 8 animals

Supplementary Table 3. Electrophysiological analysis of spontaneous and evoked synaptic transmission in wildtype, GluRIIA^{rescue}, GluRIIA^{EGFP}, GluRIIA^{ALFA} and GluRIIA^{KO} larvae (related to Figure 3). Numerical values are given as mean ± SEM for each genotype. Sample sizes for the number of NMJs and the number of animals used for analysis are indicated. For statistical comparison see Supplementary Table 4.

**genotype**	**eEPSC amplitude**	**mEPSC amplitude**	**PPR (30 ms IPI)**	**quantal content**
wildtype vs. GluRIIA^{rescue}	> 0.999	> 0.999	0.975	> 0.999
GluRIIA^{rescue}				
vs. GluRIIA^{EGFP}	> 0.999	> 0.999	0.365	> 0.999
vs. GluRIIA^{ALFA}	0.989	> 0.999	0.167	> 0.999
vs. GluRIIA^{KO}	> 0.999	< 0.001	> 0.999	0.002

Supplementary Table 4. Statistical comparison of spontaneous and evoked synaptic transmission in wildtype, GluRIIA^{rescue}, GluRIIA^{EGFP}, GluRIIA^{ALFA} and GluRIIA^{KO} larvae (related to Figure 3). p-values revealed by Ordinary one-way ANOVA for parametric data (eEPSC amplitude and PPR) or by Kruskal-Wallis test for non-parametric data (mEPSC amplitude and quantal content) are given for comparisons between wildtype and GluRIIA^{rescue} or for comparisons between GluRIIA^{rescue}, GluRIIA^{EGFP}, GluRIIA^{ALFA} and GluRIIA^{KO}, respectively.

parameter	wildtype (DMSO)	wildtype (PhTx)	$GluRIIA^{rescue}$ (DMSO)	$GluRIIA^{rescue}$ (PhTx)
**eEPSC amplitude [-nA]**	41.50 ± 3.69<br>n = 9 NMJs,<br>6 larvae	37.59 ± 3.63<br>n = 10 NMJs,<br>7 larvae	38.26 ± 3.77<br>n = 10 NMJs,<br>6 larvae	37.73 ± 2.68<br>n = 10 NMJs,<br>7 larvae
**mEPSC amplitude [-nA]**	0.655 ± 0.025<br>n = 9 NMJs,<br>6 larvae	0.433 ± 0.019<br>n = 10 NMJs,<br>7 larvae	0.697 ± 0.042<br>n = 10 NMJs,<br>6 larvae	0.482 ± 0.019<br>n = 10 NMJs,<br>7 larvae
**quantal content**	62.71 ± 4.22<br>n = 9 NMJs,<br>6 larvae	86.68 ± 11.11<br>n = 10 NMJs,<br>7 larvae	55.36 ± 5.06<br>n = 10 NMJs,<br>6 larvae	79.59 ± 6.81<br>n = 10 NMJs,<br>7 larvae

Supplementary Table 5. Electrophysiological analysis of acute presynaptic homeostasis in wildtype and $GluRIIA^{rescue}$ animals (related to Figure 3). Numerical values are given as mean ± SEM for each group. Sample sizes for the number of NMJs and the number of animals used for analysis are indicated. For information of statistical analysis see Supplementary Table 6.

genotype	eEPSC amplitude	mEPSC amplitude	quantal content
wildtype (DMSO)<br>vs. wildtype (PhTx)<br>vs. GluRIIA^{rescue}(DMSO)	 0.461<br>0.549	 < 0.001<br>0.604	 0.002<br>0.243
wildtype (PhTx)<br>vs. GluRIIA^{rescue} (PhTx)	 0.976	 0.630	 0.684
GluRIIA^{rescue} (DMSO)<br>vs. GluRIIA^{rescue} (PhTx)	 0.910	 < 0.001	 0.006

Supplementary Table 6. Statistical comparison of acute presynaptic homeostasis in wildtype and GluRIIA^{rescue} animals (related to Figure 3). p-values revealed by parametric t-test (eEPSC amplitude) or by Mann-Whitney Rank Sum tests for non-parametric data (mEPSC amplitude and quantal content) are given for comparisons between both genotypes or between measurements in DMSO and PhTx within an individual genotype, respectively.

parameter	GluRIIA^{ALFA} clusters, median (25th-75th percentile
localizations per cluster	80 (59-115)
cluster area [µm^2]	0.146 (0.010-0.208)
n (clusters, NMJs, animals)	635, 24, 10

Supplementary Table 7. *d*STORM analysis of GluRIIA^{ALFA}. Related to Figure 4.. Data were derived from analysis of the whole dataset i.e., without selecting according to AZ circularity (see Material and Methods). Non-parametric data, reported as median (25th-75th percentile).
